# Dynamic crosstalk between Tspan4^+^ macrophage subsets and MSCs via migrasomes orchestrates fracture repair

**DOI:** 10.3389/fcell.2025.1666465

**Published:** 2026-01-09

**Authors:** Siyu Zhang, Mengci Wang, Abudurexiti Kutibiding, Dandan Liu, Tuersunnayi Manafu, Wen Zhao, Yi Yang

**Affiliations:** 1 Department of Spinal Minimally Invasive and Precision Orthopedics, The First Affiliated Hospital of Xinjiang Medical University, Urumqi, China; 2 Department of Histology and Embryology, School of Basic Medical Sciences, Xinjiang Medical University, Urumqi, China

**Keywords:** migrasomes, macrophages, bone marrow mesenchymal stem cells, fracture healing, AMPK, IL1b

## Abstract

**Background:**

The cell - cell communication between macrophages and mesenchymal stromal/stem cells (MSCs) holds pivotal importance in the fracture healing process. Considering the intricate nature of the *in vivo* bone regeneration microenvironment, elucidating the changes in different macrophage subsets within this microenvironment, as well as the cell - cell communication between these subsets and MSCs, is essential for the differentiation, recruitment, and regulation of MSCs. This study was designed to investigate the interactions between diverse macrophage subsets and MSCs during the fracture healing period.

**Methods:**

Single - cell sequencing was utilized to analyze the expression of Tspan4^+^, Lyve1^+^, and Mpeg1^+^ in macrophages during fracture healing, along with the cell - interaction signals with MSCs. It was demonstrated that the cell - interaction signal transduction might be linked to migrasomes. Scratch assays and transwell assays were carried out to assess the migration capacity of MSCs affected by exosomes and migrasomes derived from Tspan4^+^Mpeg1^+^ macrophages. Micro-CT and immunofluorescence techniques were employed to observe the impacts of exosomes and migrasomes from 100 μg/mL Tspan4^+^Mpeg1^+^ macrophages on femoral fracture healing in mice.

**Results:**

Through single - cell sequencing, it was ascertained that macrophages highly expressed Tspan4 during the fracture healing process and could be categorized into Tspan4^+^Lyve1^+^ macrophages and Tspan4^+^Mpeg1^+^ macrophages. By means of cell - communication analysis, Tspan4^+^Lyve1^+^ macrophages and Tspan4^+^Mpeg1^+^ macrophages were proposed to interact with MSCs via Gas6 - Axl and IL1b - IL1r1, respectively. Collectively, macrophage-derived migrasomes convey IL-1β to MSCs to activate AMPK, thereby enhancing BMSC migration and likely osteogenic priming during fracture repair. These findings identify migrasomes as a previously underappreciated conduit in macrophage–BMSC crosstalk and suggest a vesicle-based strategy to improve fracture healing.

## 1 Introduction

Bone healing represents an exquisitely intricate and precisely orchestrated biological process, entailing a diverse array of cell types and elaborate signaling cascades (Jiang et al., 2024). Central to this process is the interaction between macrophages and bone marrow - derived mesenchymal stem cells (MSCs). As a fundamental element of the immune system, macrophages display remarkable plasticity, being capable of differentiating into distinct subsets within the bone microenvironment, each subset fulfilling unique functions (Kim et al., 2020). Simultaneously, MSCs possess the potential to differentiate into osteoblasts, which are essential for the formation of new bone during the fracture healing process.

The *in vivo* bone regeneration microenvironment is exceedingly complex, being constituted by a wide variety of cytokines, growth factors, and extracellular matrix components (Chen et al., 2023). Representative cytokines include IL-1β, TNF-α, IL-6, and chemokines such as CXCL12 that regulate recruitment, survival, and differentiation of MSCs (Chen et al., 2023). In such a complex setting, the dynamic changes in different macrophage subsets and the cell - cell communication between these subsets and MSCs are of crucial significance for regulating the differentiation, recruitment, and functionality of MSCs (Kim et al., 2020). However, due to the complexity of the *in vivo* microenvironment, our knowledge of the specific roles and mechanisms underlying these interactions remains relatively limited. Previous investigations have shown that cell communication serves as a key determinant in modulating cell behavior during bone healing. Among the diverse modalities of cell communication, the signal exchange between different cell types through extracellular vesicles, such as exosomes and migrasomes, has garnered increasing attention (Xu et al., 2023). Migrasomes, newly identified extracellular vesicles formed during cell migration, have been reported to be involved in cell - cell communication as well as the transfer of substances and information (Zhang et al., 2023; Zhang et al., 2025).

In the context of bone regeneration, although the interaction between macrophages and MSCs has been explored to some degree, the specific functions of different macrophage subsets in this interaction, especially the potential participation of migrasomes in the communication process, remain unclear. Therefore, a comprehensive exploration of the interaction between different macrophage subsets and MSCs during the fracture healing process is conducive to a more profound understanding of the regulatory mechanisms of bone regeneration.

This study aimed to investigate the interaction between different macrophage subsets and MSCs during the fracture healing process. Single-cell sequencing revealed that all macrophages express Tspan4 during fracture healing, and on the basis of uniform Tspan4 expression, these macrophages further express Lyve1 and Mpeg1 respectively.

Studies have demonstrated that LYVE1^+^ macrophages play a crucial role in early tissue repair (e.g., regulating the adipogenic potential of progenitor cells) (Justynski et al., 2023; Yu et al., 2025), while MPEG1^+^ macrophages are often associated with inflammatory clearance and antimicrobial functions during the resolution phase. These insights provide context for the distinct roles of the Tspan4^+^Lyve1^+^ and Tspan4^+^Mpeg1^+^ macrophage subsets in healing (Ba et al., 2020).

Single - cell sequencing was employed to analyze the expression of Tspan4, Lyve1, and Mpeg1 in macrophages and their interaction signals with MSCs. Moreover, scratch assays, transwell assays, Micro-CT, and immunofluorescence techniques were utilized to explore the effects of exosomes and migrasomes derived from Tspan4^+^Mpeg1^+^ macrophages on the migration of MSCs and the healing of femoral fractures in mice. This research is expected to provide novel insights into the regulatory mechanisms of bone healing and lay the groundwork for the development of new therapeutic strategies for fractures.

To our knowledge, this is the first study to investigate the role of migrasomes in macrophage–BMSC communication during fracture healing.

## 2 Materials and methods

### 2.1 Mice

All animal experiments were approved by the Laboratory of the Animal Ethics Committee of the First Affiliated Hospital of Xinjiang Medical University and were carried out in accordance with the guidelines of the National Health and Medical Research Council of China.Twenty male C57/BL6 mice with an average body weight of 20 g were obtained from Xinjiang Medical University. These mice were housed in individual cages with a 12-h light/dark cycle and were fed with standard food and water.

### 2.2 Establishment of the mouse femoral model

Mice were anesthetized intraperitoneally with 0.8% sodium pentobarbital (50 mg/kg) to provide surgical anesthesia and analgesia per IACUC protocol. They were divided into two groups: a control (same volume 10ul PBS injected at the site of fracture), and an experiment (100ug/mL Migrasomes from Tspan4+Mpeg1+ RAW264.7 injected at the site of fracture) (ten in each group). The outer side of the thigh was prepared for shaving, fixed in a lateral decubitus position, and routinely disinfected on the outer side of the thigh. A 5-mm longitudinal incision was made between the right knee joint and the hip joint under aseptic conditions. After blunt dissection to expose the femoral shaft on one side of the mouse femur, the femur in the middle of the femoral shaft was cut off. The intercondylar groove of the femur was fully flexed at the knee joint to expose it, and a drill hole with a diameter of 0.5 mm was made in the center of the intercondylar groove. A 24-gauge needle with a diameter of 0.5 mm was inserted into the drill hole in the center of the intercondylar groove, and the tip of the needle penetrated through the top of the greater trochanter of the femur. After successfully establishing the fracture model, local disinfection was carried out using povidone-iodine, and then the surgical skin incision was sutured layer by layer. After disinfecting the wound again with povidone-iodine, it was marked and returned to the mouse cage.

### 2.3 Data collation

In the Gene Expression Omnibus (GEO, https://www.ncbi.nlm.nih.gov/geo/) database, the single-cell RNAseq dataset related to the mouse fracture model, numbered GSE192630, was retrieved and obtained. We aligned this public dataset with our *in vivo* model for downstream analyses.

In the R 4.2.2 software, the Seurat, decontX, and scDblFinder software packages were used to read, perform quality control, and integrate the data of the above dataset. The quality control conditions were as follows: the total number of genes detected in each cell was between 200 and 7,000, the total number of transcript molecules (unique molecular identifiers, UMI) was greater than 10, and the proportion of mitochondrial genes was less than 15%; the proportion of environmental RNA contamination by decontx was less than 0.2; scDblFinder judged it as a single cell. Cells meeting the above conditions were retained for subsequent analysis. Then, combined with the harmony software package, batch effect correction was carried out on the integrated data.

Finally, using the markers of known cells, cell type identification was carried out. The markers are as follows:

Macrophages: “Adgre1”, “Msr1”;
Monocytes: “Cd14”; Dendritic cells: “Flt3″, “Itgax”;
Neutrophils: “Ngp”, “Elane”, “S100a8”; Endothelial cells: “Pecam1″, “Eng”, “Erg”; Bone marrow mesenchymal stem cells: “Prrx1″, “Pdgfra”, “Ly6a”, “Runx2″, “Sp7″, “Thy1”;
Osteocytes: “Acp5″, “Ctsk”, “Ocstamp”;
Fibroblasts: “Tagln”, “Tpm2”; Erythroid cells: “Alas2″, “Hba-a2”;
Schwann cells: “Sox10″, “S100b”.

And cell proportion diagrams of annotation results, t-SNE diagrams, and mean heatmaps of cell markers were drawn.

### 2.4 Analysis of macrophage subtypes and ligand-receptor pairs

In the R 4.2.2 software, the Seurat software package was used to extract macrophages from the above cells for re-integration and clustering analysis, and two groups of distinct macrophages were obtained: Lyve1^+^ macrophages and Mpeg1^+^ macrophages.

The CommPath software package was used to analyze the ligand-receptor pairs of the interaction changes between macrophage subsets and bone marrow mesenchymal stem cells. Based on the time changes and expression trends, two pairs of ligand-receptor pairs, Gas6/Axl and Il1b/Il1r1, were selected for subsequent analysis.

Combined with the KEGG database, the signaling pathways in the mouse pathways where these two pairs of ligand-receptor pairs appeared simultaneously were selected as the potential signaling pathways for these two pairs of ligand-receptor pairs, and the associations were visualized.

Using the gsva algorithm of the GSVA software package, combined with migrasome-related markers such as “Ndst1”, “Eogt”, “Pigk”, “Cpq”, “Tspan4”, “Tspan7”, etc., the potential occurrence of migrasomes in macrophages was evaluated.

### 2.5 Immunofluorescence staining

Mouse fracture callus tissues on days 0, 5, and 10 post-fracture were harvested for immunofluorescence analysis. Tissues were fixed in 4% paraformaldehyde and decalcified, then cryosectioned. Sections were blocked and incubated with primary antibodies against LYVE1, MPEG1, F4/80 (Adgre1), and Tspan4, followed by appropriate fluorophore-conjugated secondary antibodies. Nuclei were counterstained with DAPI. Stained sections were observed and imaged under a confocal fluorescence microscope to visualize the localization of macrophage marker.

### 2.6 Detection of the ability of migrasomes derived from Tspan4^+^Mpeg1^+^ macrophages to promote fracture healing in the mouse femoral fracture model

Ten mice were subjected to the same fracture model as described above. When the mice underwent the above fracture model, they were divided into the migrasome injection group at the fracture site and the fracture blank control group (n = 5/group). Take 50 μg of the successfully extracted migrasome sample, dilute it to 200 μL with diluentc (PBS) respectively, and mix it evenly; transfer 200 μL of the migrasome sample. Then added to 800 μL of a 25% (w/v) thermosensitive hydrogel (Pluronic F-127) solution with thorough mixing. The resulting hydrogel–migrasome suspension was then injected *in situ* at the fracture site.

### 2.7 Micro-CT evaluation of fracture healing in a mouse femoral fracture model treated with Tspan4^+^Mpeg1^+^ macrophage-derived migrasomes

Micro-CT was performed on the 14thday after the surgical treatment. The mice were anesthetized, and the position of the left femur of the mice was fully exposed. The small animal Micro-CT imaging system produced by Shanghai Yuyan Scientific Instrument Co., Ltd. was used to scan the femur of the mice. The Micro-CT scanning parameters were as follows: image matrix 1024 × 1024, integration time 4 min, energy/intensity 90 kVp, 100 μA, 8 W, resolution 18 μm, rotation scanning at 0°, and the scanning range was set as the upper middle segment and the middle lower segment of the femur. After the scanning was completed, the cancellous bone tissue at a position 1.0 mm distal to the growth plate of the longitudinal section of the femur with a layer thickness of 1.0 mm was selected as the region of interest for three-dimensional reconstruction and quantitative analysis, and the minimum threshold of the image information was 170.

### 2.8 Cell culture

The bone marrow MSCs (Procell, Cat. No. CP-M131, Wuhan, China) were cultured in MEM with 10% FBS at 37 °C in 5% CO_2_. The identity of these MSCs was confirmed by immunofluorescence staining for the surface marker CD44, with over 90% of cells testing positive (Identification Report No. JDBG240490, iCell Bioscience, Shanghai). The successfully transfected Tspan4^+^Mpeg1^+^ macrophages were cultured in DMEM supplemented with 10% FBS (Thermo).

### 2.9 Plasmid construction and macrophage transfection

Exploration of puromycin (Puro) drug killing: RAW264.7 cells were seeded in a 24-well plate, 1 × 10^5^ cells per well, and 500 μL of complete medium was added to each well and cultured overnight. The medium containing puromycin at concentrations of 0, 0.5, 1, 2, 3, 4, 5, 6, 7, 8, 9, 10 μg/mL was replaced, with two duplicate wells for each concentration. After adding the drug for 24 h, the medium containing the corresponding puromycin was replaced; after 48 h, cell survival was monitored, and 500 μL of puromycin-free medium was added; the puromycin concentration in the experimental group where no cells survived after 72 h was the screening concentration, which was 2 μg/mL. RAW264.7 macrophages were transfected with lentiviral plasmids carrying the coding sequences (CDS) of mouse Tspan4 (732 bp) and Mpeg1 (2187 bp). The Tspan4 CDS was synthesized and cloned into the pLV4ltr-PGK-ZsGreen(2A) PURO-CMV vector (Amp^R backbone) using XhoI/BamHI sites, and the Mpeg1 CDS into the pLV-mCherry(2A) BSD-CMV vector (Amp^R, XhoI/BamHI). One day before transfection, RAW264.7 cells (∼80% confluency) were prepared in 24-well plates. Plasmid DNA and Lipo293™ transfection reagent were mixed according to the manufacturer’s protocol (approx. MOI 60 for lentiviral particles) and added to the cells. 24 h after transfection, selection antibiotics were applied to establish stable cell lines: 2 μg/mL puromycin for Tspan4-transfected cells and 5 μg/mL blasticidin for Mpeg1 (co-transfected) cells. Selection was maintained for 48 h, after which cells were returned to antibiotic-free medium. Successful overexpression was confirmed by qRT-PCR for Tspan4 and Mpeg1 mRNA and by fluorescence microscopy (ZsGreen and mCherry signals in transfected cells).

#### 2.9.1 Plasmid construction and macrophage transfection

Gene source and length: Tspan4 CDS = 732 bp and Mpeg1 CDS = 2187 bp were gene-synthesized and delivered as plasmid DNA plus glycerol stocks.

Vectors and cloning sites: Inserts were subcloned using XhoI/BamHI into pLV4ltr-PGK-ZsGreen(2A)PURO-CMV for Tspan4 and pLV-mCherry(2A)BSD-CMV for Mpeg1 (Amp^R backbones). Correct insertion and orientation were confirmed by dual-digest and Sanger sequencing.

Transfection/build strategy: To generate Tspan4^+^ and Tspan4^+^Mpeg1^+^ RAW264.7 cells, we transfected the Tspan4 plasmid alone or co-transfected (1:1, w/w) Tspan4 + Mpeg1 plasmids using Lipo293, followed by puromycin 2 μg/mL selection.

Reporters/selection: The vectors harbor ZsGreen-2A-Puro or mCherry-2A-BSD elements for fluorescence tracking and antibiotic markers; in this study, positivity was primarily established by dual fluorescence and qRT-PCR, while puromycin selection was used operationally to enrich transfectants.

### 2.10 Extraction and purification of migrasomes

The purification and extraction of migrasomes were carried out according to the literature (Zhang et al., 2023). The Tspan4^+^Mpeg1^+^ cells seeded on the fibronectin-coated culture dish were cultured overnight, and then the medium was discarded, and the cells were washed with PBS. Trypsin digestion was carried out, and after timing for 5 min, the cells were collected in a 15-mL centrifuge tube. Centrifuge at 1000 *g* for 10 min to remove the cell bodies, then centrifuge at 4000 *g* for 20 min to remove the cell debris, and then centrifuge at 18,000 *g* for 40 min to collect the crude migrasomes. The precipitate containing crude Migrasomes was resuspended in 800 μL of sample buffer (400 μL of 10% OptiPrep +400 μL of extraction buffer), and fractionated at 150,000×g for 4 h in an OptiPrep density gradient using an MLS50 rotor (Optima MAX-XP). The gradient was: 5% (500 μL), 10% (sample, 800 μL), 15% (500 μL), 20% (500 μL), 25% (500 μL), 30% (500 μL), 35% (500 μL), 40% (500 μL), 50% (500 μL). The fractions were prepared for negative staining, Western blot analysis, BCA assay, or RNA extraction. After successful extraction, confocal WGA staining and electron microscopy were used to identify the successful extraction.

### 2.11 Transmission electron microscopy

For the treated cells, discard the medium, wash with PBS to remove the residual medium, add 2.5% glutaraldehyde and fix at room temperature for 4 h. Gently scrape off the cells with a cell scraper and transfer them into an EP tube. Then, rinse with 0.1M phosphate buffer PBS (PH7.4) three times, 15 min each time. Fix with 1% osmium tetroxide prepared with 0.1M phosphate buffer PBS (PH7.4) in the dark at room temperature for 2 h. Rinse with 0.1M phosphate buffer PBS (PH7.4) three times, 15 min each time. The tissue was successively immersed in 30%–50%–70%-80%–95%–100%–100% alcohol for upward dehydration, 20 min each time, twice with 100% acetone, 15 min each time. Acetone: 812 embedding agent = 1:1 at 37 °C for 2–4 h, acetone: 812 embedding agent = 1:2 at 37 °C for overnight penetration, and pure 812 embedding agent at 37 °C for 5–8 h. Pour the pure 812 embedding agent into the embedding plate, insert the sample into the embedding plate, and place it in an oven at 37 °C overnight. Put the embedding plate in an oven at 60 °C for polymerization for 48 h, and take out the resin block for later use. Ultrathin sectioning: The resin block was ultrathin sectioned at 60–80 nm with an ultramicrotome, and the sections were picked up with a 150-mesh Formvar film copper mesh. Stain the copper mesh with a saturated alcohol solution of 2% uranyl acetate in the dark for 8 min; wash with 70% alcohol three times; wash with ultrapure water. Stain with a 2.6% lead citrate solution to avoid carbon dioxide for 8 min; wash with ultrapure water three times, and slightly dry with filter paper. Put the copper mesh sections into a copper mesh box and dry at room temperature overnight. Observe under a transmission electron microscope (HITACHI, HT7800/HT7700) and collect images.

### 2.12 Transwell

Resuspend the cells with serum-free MEM medium and add them to the Transwell chamber of a 24-well plate. The lower chamber was filled with 20% FBS-containing medium plus 100 ng/mL migrasomes derived from Tspan4^+^Mpeg1^+^ macrophages. The control group received the same 20% FBS medium without migrasomes (volume replaced with PBS). Incubate in an incubator at 37 °C with 5% CO_2_ for 24 h; take out the Transwell, wipe off the cells on the inner layer of the membrane, stain with 0.5% crystal violet staining solution for 20 min, wipe off the non-migrated cells on one side of the upper chamber, and observe and take photos under a microscope.

### 2.13 Scratch assay

Seed MSCs at a density of 3 × 10^5^ in a six-well plate. When the confluence reaches 80%, make a scratch with a 200-μL pipette tip. After making the scratch, discard the original medium. Add the medium containing 100 ng/mL migrasomes from Tspan4^+^Mpeg1^+^ macrophages to the experimental group, and add the same volume of PBS to the control group. Take photos and record the photos at 0 h, and then take photos and record them after 24 h.

### 2.14 Western blot

Prepare the tissue lysis buffer with PMSF:RIPA = 1:100, and operate on ice. Discard the cell culture medium, add 150 μL/well of the tissue lysis buffer to lyse the cells, and lyse them fully in the dark for 30 min as timed. Scrape off the cells with a cell scraper and put them into a labeled 2-mL EP tube, and place it on ice. The subsequent steps are the same: after centrifugation, aspirate the supernatant into a new labeled EP tube. After protein quantification with the BCA kit, boil the protein (100 °C for 10 min). Prepare the SDS-PAGE gel, perform electrophoresis, transfer the protein to the PVDF membrane, block with 5% skim milk for 2 h, incubate with the primary antibodies: IL-1β (1:3000), AMPK (1:2000) overnight at 4 °C, wash with 1X TBST, incubate with the secondary antibody at room temperature for 2 h, wash with 1X TBST, immerse the PVDF membrane in the ECL luminescent solution (mix solution A and solution B at a ratio of 1:1), and develop and expose. Quantify the protein bands with ImageJ.

## 3 Results

### 3.1 Identification and dynamic changes of macrophage subsets during fracture healing in mice

**FIGURE 1:**
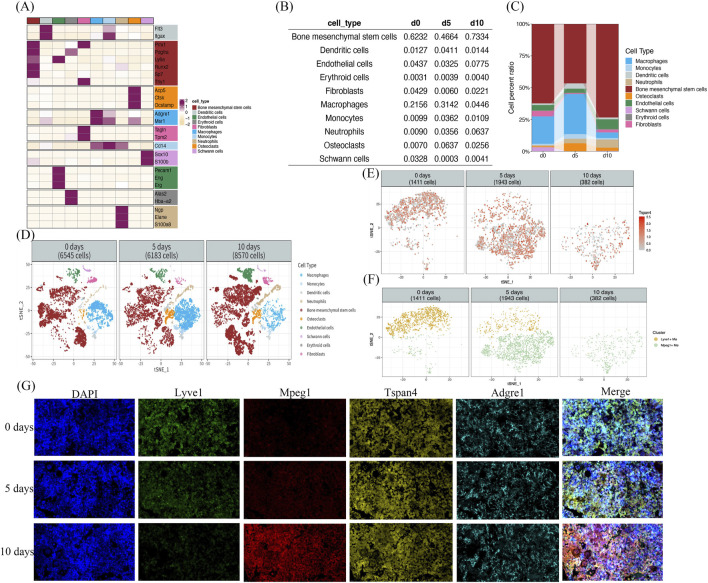
Identification and dynamic changes of macrophage subsets during fracture healing in mice. **(A–D)** Known cell markers were used to identify cell types; cell proportion plots, t-SNE plots of the annotated data, and heatmaps of mean cell marker expression values were generated. **(E)** Single-cell transcriptomic analysis revealed changes in Tspan4 expression in macrophages during fracture healing. **(F)** Integrated clustering of macrophages identified two distinct subsets: Lyve1^+^ and Mpeg1^+^ macrophages. **(G)** Immunofluorescence images of LYVE1 (green), MPEG1 (red), the pan-macrophage marker Adgre1 (cyan), and Tspan4 (yellow) in fracture callus samples on days 0, 5, and 10 post-fracture (merged with DAPI; scale bar: 10 μm).

The single - cell RNA - sequencing dataset associated with the mouse fracture model, bearing the accession number GSE192630, was retrieved from the Gene Expression Omnibus database. In the R 4.2.2 environment, the Seurat, decontX, and scDblFinder software packages were utilized to read, execute quality control procedures, and integrate the data of the aforementioned dataset. By leveraging markers of known cell types, cell type identification was carried out. Subsequently, cell proportion diagrams based on the annotation outcomes, t-SNE diagrams, and mean heatmaps of cell markers were constructed (Figures 1A–D). Figures 1A–D summarizes QC and annotation to validate cell identities prior to subset analyses; days 0, 5, and 10 correspond to injured baseline, early callus, and mid-healing, respectively. Notably, in the t-SNE plot (Figure 1B), each numeric label denotes a distinct cell cluster identified in our analysis (for example, cluster “1” comprises Lyve1^+^ macrophages and cluster “2” comprises Mpeg1^+^ macrophages). Additionally, the heatmap of mean marker expression (Figure 1D) shows that MSCs are characterized by high expression of osteogenic markers, whereas the macrophage subsets segregate according to their Tspan4, Lyve1, and Mpeg1 expression profiles, reflecting distinct functional identities.

Through the analysis of macrophage gene expression, it was revealed that all macrophages within the samples exhibited high - level expression of Tspan4 (Figure 1E). Employing the Seurat software package, macrophages were isolated from the previously identified cell population for re - integration and clustering analysis. Consequently, two distinct groups of macrophage subsets were obtained: Lyve1^+^ macrophages and Mpeg1^+^ macrophages.

We identified two distinct macrophage subsets (Tspan4^+^Lyve1^+^ and Tspan4^+^Mpeg1^+^). The Tspan4^+^Lyve1^+^ subset progressively decreased from day 0 to day 10, whereas the Tspan4^+^Mpeg1^+^ subset expanded to a peak at day 5 and then partially declined by day 10 (Figures 1E–G). Using Adgre1 as a pan-macrophage marker, immunofluorescence staining on fracture callus tissue (days 0, 5, 10) confirmed that all Adgre1^+^ macrophages express Tspan4 and can be subdivided into Tspan4^+^Lyve1^+^ and Tspan4^+^Mpeg1^+^ subsets. Moreover, the immunofluorescence results mirrored the single-cell data: the Tspan4^+^Mpeg1^+^ macrophages increased from day 0 to day 5 and then declined by day 10, whereas the Tspan4^+^Lyve1^+^ macrophages steadily decreased over this period (Figures 1F,G).

These findings prompted us to hypothesize the emergence of a new balance among macrophage subsets as fracture healing progresses. By the mid-healing stage (day 10), the initially prevalent Tspan4^+^Lyve1^+^ subset had markedly waned, and even the Tspan4^+^Mpeg1^+^ subset—after peaking at day 5 — showed a partial decline, indicating a re-balancing of macrophage populations in the later stage of healing. Nevertheless, the pronounced expansion of the Tspan4^+^Mpeg1^+^ subset (with a peak at day 5) suggests that this subset is a principal macrophage population involved in regulating crosstalk with MSCs during fracture repair.

### 3.2 Cell communication occurs between Tspan4^+^Lyve1^+^ macrophages/Tspan4^+^Mpeg1^+^ macrophages and MSCs via Gas6 - Axl and IL1b - IL1r1 pathways respectively

**FIGURE 2:**
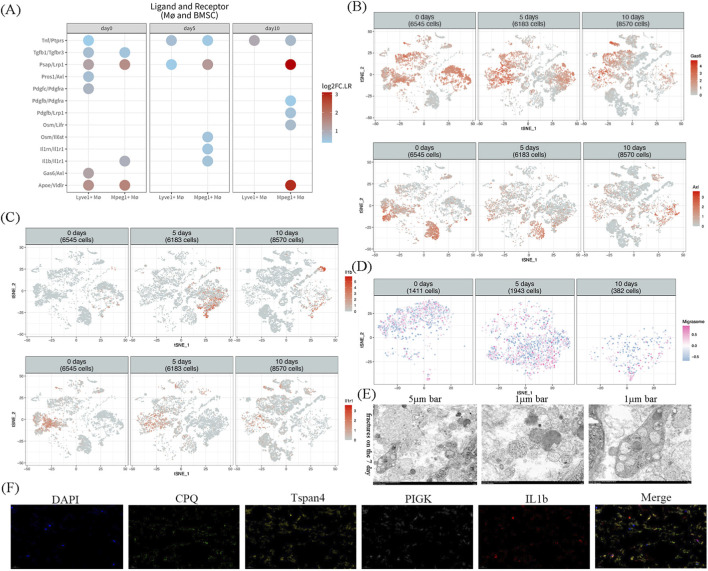
Tspan4^+^Lyve1^+^ and Tspan4^+^Mpeg1^+^ macrophages communicate with bone marrow mesenchymal stem cells (MSCs) via the Gas6–Axl and IL-1β–IL-1R1 pathways, respectively. **(A)** The CommPath analysis of ligand–receptor interactions between macrophage subsets and MSCs identified two key signaling pairs, Gas6/Axl and Il1b/Il1r1, based on their temporal expression trends. **(B,C)** Single-cell transcriptomic data showing the expression dynamics of Gas6 and Il1b in macrophages and Axl and Il1r1 in MSCs during fracture healing. **(D)** GSVA analysis using migrasome-related markers (e.g., Ndst1, Eogt, Pigk, Cpq, Tspan4, Tspan7). to evaluate the potential involvement of migrasome formation in macrophages. **(E)** Migrasome structures observed by transmission electron microscopy in day-7 fracture samples (scale bars: 5 μm and 1 μm). **(F)** Immunofluorescence images of CPQ (green), IL-1β (red), PIGK (white), and Tspan4 (yellow) in day-7 fracture callus (merged with DAPI; scale bar: 20 μm).

To explore the interaction relationships among the aforementioned distinct macrophage subsets and MSCs during fracture healing, we utilized the CommPath software package to independently analyze the ligand - receptor pairs involved in the interaction alterations between Tspan4^+^Lyve1^+^ macrophages, Tspan4^+^Mpeg1^+^ macrophages, and MSCs. We found that Gas6/Axl signaling steadily declined over time, whereas Il1b - Il1r1 signaling increased during fracture healing. This observation led us to speculate that Gas6–Axl signaling predominantly supports the early phase of healing, whereas Il1b - Il1r1 signaling via migrasomes from Tspan4^+^Mpeg1^+^ macrophages become more crucial in later stages, promoting sustained MSC migration and osteogenic differentiation. (Figure 2A). Furthermore, during the fracture healing process, as the fracture healed, the expression of Gas6 in Tspan4^+^Lyve1^+^ macrophages and that of Axl in MSCs steadily declined (Figure 2B). In contrast, the expression of IL1b in Tspan4^+^Mpeg1^+^ macrophages and the expression of IL1r1 in MSCs increased consistently throughout the fracture healing process (Figure 2C).

Notably, Tspan4 has been reported as one of the crucial genes involved in migrasome production. Recent research has shown that when monocytes are stimulated by lipopolysaccharide (LPS), a substantial amount of inflammatory cytokines within monocytes are conveyed into migrasomes through secretory carriers (Jiao et al., 2024). This process culminates in the extracellular release of inflammatory factors, which are shed from isolated migrasomes (Jiao et al., 2024). This discovery prompts us to hypothesize that macrophages with high Tspan4 expression in the fracture microenvironment could carry out signal transduction via migrasomes, a newly identified vesicular organelle. Utilizing the gsva algorithm within the GSVA software package, in conjunction with migrasome - related markers such as “Ndst1″, “Eogt”, “Pigk”, “Cpq”, “Tspan4″, and “Tspan7″, we assessed the potential formation of migrasomes in macrophages (Figure 2D) (Zhao et al., 2019). Our findings suggest that macrophage - derived migrasomes may be linked to the transfer of IL1b (Figure 2D). By means of transmission electron microscopy to observe the tissue samples of mouse fractures on the 7th day, we uncovered that a large number of migrasomes were present during the fracture healing process in mice (Figure 2E). Through immunofluorescence staining of PIGK, CPQ, Tspan4, and IL1b in mouse fractures on the 7th day, we detected that IL1b was expressed on small vesicles approximately 2 μm in size, which also expressed PIGK, CPQ, and Tspan4. As migrasomes are extracellular, DAPI signals from nuclei do not co-localize with migrasome puncta, co-staining is shown only to provide tissue context.This further implies that the cell - cell interaction between Tspan4+Mpeg1+ macrophages and MSCs via the IL1b - IL1r1 pathway might be mediated by migrasomes (Figure 2F).

### 3.3 Migrasomes from Tspan4^+^Mpeg1^+^ macrophages mediate the regulation of the migration ability of MSCs through the IL1b-IL1r1 pathway

**FIGURE 3:**
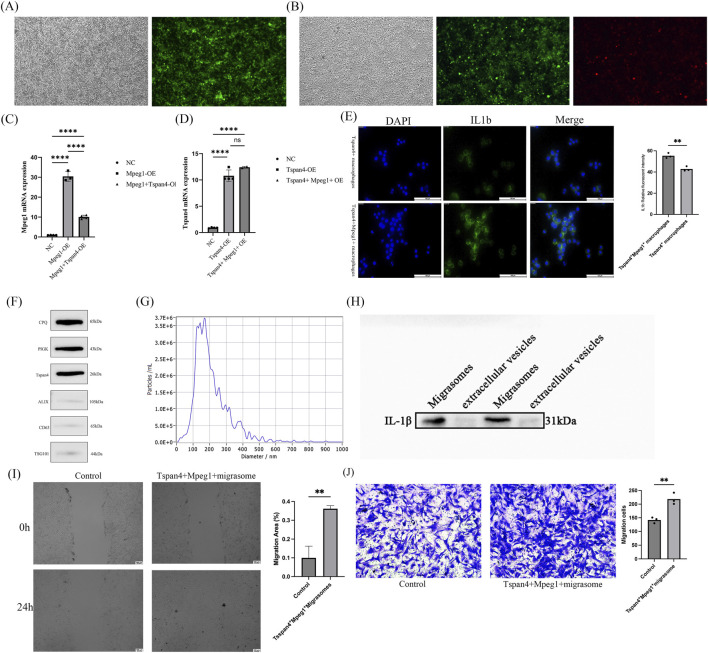
Migrasomes of Tspan4^+^ Mpeg1^+^ macrophages mediate the regulation of the migration ability of MSCs through the IL1b-IL1r1 pathway. **(A,B)** Observe the transfection efficiency of Tspan4^+^ macrophages and Tspan4^+^Mpeg1^+^ macrophages using a confocal fluorescence microscope. **(C)** Detect the expression of Mpeg1 mRNA by qRT-PCR. **(D)** Detect the expression of Tspan4 mRNA by qRT-PCR. **(E)** Perform IL1b immunofluorescence staining on Tspan4^+^ macrophages and Tspan4^+^Mpeg1^+^ macrophages. The scale bar is 10 μm. **(F)** Detect the expression of Tspan4, PIGK, CPQ, and Alix in migrasomes by Western blot. **(G)** Detect the particle size of the extracted extracellular vesicles by NTA. **(H)** Detect the expression of IL1b in extracellular vesicles and migrasomes by Western blot. **(I)** Co-culture the extracellular vesicles and migrasomes of Tspan4^+^Mpeg1^+^ macrophages with MSCs at a concentration of 100 ng/mL respectively, and detect the migration ability of MSCs 24 h after co-culture by the wound healing assay. *p < 0.05. **(J)** Co-culture the extracellular vesicles and migrasomes of Tspan4+Mpeg1+ macrophages with MSCs (100 ng/mL each), and assess MSC migration after 24 h by the Transwell assay. Data represent mean ± SD of three independent experiments. *p < 0.05.

To further explore the mechanism of action of Tspan4^+^Mpeg1^+^ macrophages in the fracture microenvironment, we constructed Tspan4^+^ macrophages and Tspan4^+^Mpeg1^+^ macrophages *in vitro* using plasmids. The expressions of Tspan4 and Mpeg1 were detected by confocal microscopy and qRT - PCR to confirm the successful construction of Tspan4^+^ macrophages and Tspan4^+^Mpeg1^+^ macrophages *in vitro* (Figures 3A–D). Immunofluorescence was used to detect IL-1β expression in Tspan4^+^ and Tspan4^+^Mpeg1^+^ macrophages, and we found that Tspan4^+^Mpeg1^+^ macrophages had a significantly higher expression of IL-1β than Tspan4^+^ macrophages (Figure 3E). Since previous data indicated that the cell - cell interaction between Tspan4^+^Mpeg1^+^ macrophages and MSCs via the IL - 1β - IL - 1R1 axis might be mediated by migrasomes, we extracted migrasomes and extracellular vesicles derived from Tspan4^+^Mpeg1^+^ macrophages. Western blot (WB) analysis showed that the samples highly expressed migrasome - marker proteins CPQ, PIGK, and Tspan4, while the expressions of ALIX, CD63, and TSG101 were low, indicating successful extraction of migrasomes (Figure 3F). The particle size of the extracted extracellular vesicles was detected by nanoparticle tracking analysis (NTA), and the results showed that the particle size was distributed at around 200 nm, demonstrating successful extraction of small extracellular vesicles (Figure 3G). WB was used to detect the expression of IL - 1β in small extracellular vesicles and migrasomes, and it was found that the expression of IL - 1β was higher in migrasomes than in small extracellular vesicles (Figure 3H). This indicates that the signal transduction of Tspan4^+^Mpeg1^+^ macrophages through IL - 1β is mediated by migrasomes.

To further investigate the regulatory effect of migrasomes derived from Tspan4^+^Mpeg1^+^ macrophages on MSCs within the fracture microenvironment, we co - cultured the successfully isolated migrasomes from Tspan4^+^Mpeg1^+^ macrophages with MSCs at a concentration of 100 ng/mL. The scratch-wound assay demonstrated that migrasomes from Tspan4^+^Mpeg1^+^ macrophages significantly enhanced the migratory capacity of MSCs (Figure 3I). Consistently, the Transwell migration assay showed a greater number of MSCs migrated in the presence of Tspan4^+^Mpeg1^+^ macrophage-derived migrasomes than in controls (Figure 3J).

### 3.4 Migrasomes from Tspan4+Mpeg1+ macrophages regulate the expression of AMPK on MSCs, thereby modulating the migration ability of MSCs

**FIGURE 4:**
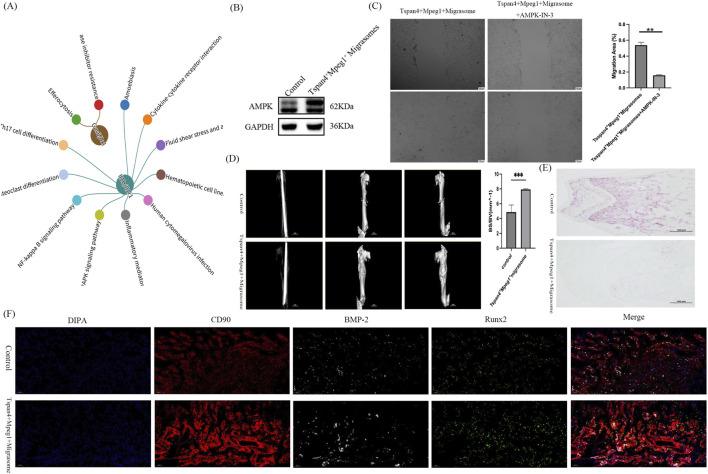
Migrasomes of Tspan4+ Mpeg1+ macrophages regulate the expression of AMPK on MSCs, thereby regulating the migration ability of MSCs. **(A)** Using the KEGG database, we identified mouse signaling pathways where the IL-1β–IL-1R1 and Gas6–Axl ligand-receptor pairs co-occur, and visualized these associations, highlighting that the IL-1β–IL-1R1 pair is linked to activation of the AMPK signaling pathway. **(B)** Detect the expression of AMPK after co-culturing migrasomes of Tspan4+Mpeg1+ macrophages at a concentration of 100 ng/mL with MSCs by Western blot. **(C)** After inhibiting AMPK with AMPK-IN-3, detect the migration ability of MSCs co-cultured with migrasomes of Tspan4+Mpeg1+ macrophages at a concentration of 100 ng/mL for 24 h by the wound healing assay. *p < 0.05. **(D)** Micro-CT was used to compare fracture healing at 14days post-fracture in mice that received an injection of migrasomes derived from Tspan4^+^Mpeg1^+^ macrophages and those in the control group. **(E)** Double staining of ALP and TRAP in bone tissue was used to compare the fracture healing status at 14 days post-fracture between mice injected with migrasomes derived from Tspan4^+^Mpeg1^+^ macrophages and control mice, and the scale bar is 500 μm. **(F)** After treatment with migrasomes of Tspan4+Mpeg1+ macrophages, perform immunofluorescence staining of RUNX2 (green), CD90 (red), and BMP-2 (white) in the samples of mouse fractures on day 10. This figure is a merged image including nuclear staining (DAPI), and the scale bar is 50 μm.

To further explore the regulatory mechanism of migrasomes from Tspan4^+^Mpeg1^+^ macrophages on MSCs in the fracture microenvironment, we integrated with the KEGG database and selected the signaling pathways in the mouse pathways where the IL1b-IL1r1 ligand-receptor pair appeared simultaneously as the potential signaling pathways for this IL1b-IL1r1 ligand-receptor pair, and visualized the associations (Figure 4A). We found that Tspan4+Mpeg1+ macrophages might regulate the AMPK signaling pathway in MSCs through IL1b-IL1r1 (Figure 4A). Pathway inference integrated KEGG co-occurrence linking IL-1β–IL-1R1 with AMPK modules, which we validated by WB and by pharmacological inhibition with AMPK-IN-3 that abrogated migrasome-induced BMSC migration. Moreover, the Western blot (WB) experiment revealed that the expression of AMPK in MSCs increased 24 h after the intervention with migrasomes from Tspan4^+^Mpeg1^+^ macrophages (Figure 4B). AMPK-IN-3 is a potent and selective AMPK inhibitor that does not affect cell viability. The scratch assay showed that in the presence of AMPK-IN-3, the ability of migrasomes from Tspan4^+^Mpeg1^+^ macrophages to induce the migration of MSCs was inhibited (Figure 4C). This also indicates that migrasomes from Tspan4^+^Mpeg1^+^ macrophages regulate the migration ability of MSCs by modulating the expression of AMPK.

To better mimic the complexity of the *in vivo* bone regeneration microenvironment, we established a mouse femoral fracture model and injected 100 μg of migrasomes derived from Tspan4^+^Mpeg1^+^ macrophages *in situ*. Micro - CT analysis demonstrated that migrasomes from Tspan4^+^Mpeg1^+^ macrophages enhanced the bone surface to bone volume (BS/BV) ratio at the fracture site and expedited fracture healing (Figure 4D). Double staining for ALP and TRAP on samples collected at day 14 of fracture healing revealed that migrasomes derived from Tspan4^+^Mpeg1^+^ macrophages reduced the number of TRAP^+^ cells while increasing the number of ALP^+^ cells during bone healing, thereby facilitating fracture repair (Figure 4E). CD90 serves as one of the markers for MSCs. On the 10th day of fracture healing, immunofluorescence staining for CD90, RUNX2, and BMP - 2 was carried out on the tissue samples (Figure 4E). It was discovered that migrasomes from Tspan4^+^Mpeg1^+^ macrophages could recruit MSCs more efficiently (Figure 4F).

## 4 Discussion

Compared with previous studies, this study, for the first time, elaborated in detail the dynamic changes of Tspan4^+^ macrophage subsets during fracture healing and their interaction mechanisms with MSCs. This enriches our understanding of cell–cell communication in fracture healing. Moreover, our findings reveal the important role of migrasomes in macrophage–MSC communication, providing a new perspective for intercellular communication in bone regeneration.

This study comprehensively explored the interactions between different macrophage subsets and bone marrow - derived mesenchymal stem cells (MSCs) during the fracture healing process. By conducting single - cell sequencing analysis, we determined that, during the fracture healing process in mice, macrophages all highly expressed Tspan4 and could be further classified into two subsets: Tspan4^+^Lyve1^+^ macrophages and Tspan4^+^Mpeg1^+^ macrophages. The number of Tspan4^+^Mpeg1^+^ macrophages rose from day 0 to day 5 and then partially declined by day 10, suggesting a transient enrichment consistent with a temporally restricted role during mid-healing. On the 0th, 5th, and 10th days post - fracture, whereas the number of Tspan4^+^Lyve1^+^ macrophages gradually decreased. This dynamic change indicates the existence of a new balance relationship among macrophages during the fracture healing process. The results of immunofluorescence staining further validated these findings and suggested that Tspan4^+^Mpeg1^+^ macrophages might be one of the key macrophage subtypes involved in regulating cell communication with MSCs.

In terms of the cell communication mechanism, we discovered that Tspan4^+^Lyve1^+^ macrophages and Tspan4^+^Mpeg1^+^ macrophages could interact with MSCs via Gas6 - Axl and IL1b–IL1r1 pathways, respectively, during fracture healing (with Gas6/Axl signaling decreasing over time and IL1b/IL1r1 signaling increasing). We speculate that Gas6–Axl signaling by Tspan4^+^Lyve1^+^ macrophages predominantly supports early fracture healing (e.g., initial BMSC recruitment or survival), whereas IL1b–IL1r1 signaling via migrasomes from Tspan4^+^Mpeg1^+^ macrophages becomes more crucial in later stages, promoting sustained BMSC migration and osteogenic differentiation. Notably, as a crucial gene for migrasome formation, Tspan4, along with the observation of numerous migrasomes during the fracture healing process by transmission electron microscopy and the analysis based on migrasome - related markers, led us to hypothesize that macrophages might perform signal transduction through migrasomes. The results of immunofluorescence staining further corroborated this hypothesis, suggesting that the cell - cell interaction between Tspan4^+^Mpeg1^+^ macrophages and MSCs via the IL1b - IL1r1 pathway could be mediated by migrasomes.

Further experiments revealed that migrasomes derived from Tspan4^+^Mpeg1^+^ macrophages could effectively enhance the migration ability of MSCs, as confirmed by both the scratch assay and the Transwell assay. Mechanistic investigations indicated that Tspan4^+^Mpeg1^+^ macrophages might regulate the AMPK signaling pathway in MSCs via the IL1b - IL1r1 axis. Additionally, Western blot analyses demonstrated that the expression of AMPK in MSCs was upregulated following intervention with migrasomes from Tspan4^+^Mpeg1^+^ macrophages. When the AMPK inhibitor AMPK - IN - 3 was applied, the capacity of migrasomes from Tspan4^+^Mpeg1^+^ macrophages to induce BMSC migration was suppressed, suggesting that migrasomes from Tspan4^+^Mpeg1^+^ macrophages modulate the migration ability of MSCs by regulating AMPK expression. Moreover, in a mouse femoral fracture model, in - situ injection of migrasomes derived from Tspan4^+^Mpeg1^+^ macrophages could more efficiently recruit MSCs, providing crucial experimental evidence for mimicking the *in vivo* bone regeneration microenvironment.

Compared with previous studies, this study, for the first time, elaborated in detail the dynamic changes of Tspan4^+^ macrophage subsets during the fracture healing process and their interaction mechanisms with MSCs. This enriches our understanding of cell communication during fracture healing. On the other hand, it revealed the important role of migrasomes in the communication between macrophages and MSCs, providing a new perspective for the study of intercellular communication in the field of bone regeneration. Although previous studies have focused on the interaction between macrophages and MSCs, the specific roles of macrophage subsets and the signal - transduction mechanisms mediated by migrasomes have remained unclear. This study has filled this gap to a certain extent.

However, this study has several limitations. First, although we observed pro-migratory effects of Tspan4^+^Mpeg1^+^ macrophage-derived migrasomes on MSCs, the specific cargo molecules and their precise impact on the AMPK pathway remain unclear. Further in-depth investigation—for example, measuring AMPK activation at additional time points—is required. Specifically, direct confirmation of the IL1b–IL1r1 signaling axis (for instance, through IL1r1 inhibition experiments) is needed to validate its role in AMPK pathway regulation. Second, this study primarily focused on the interaction between migrasomes from Tspan4^+^Mpeg1^+^ macrophages and MSCs. The comprehensive roles of other macrophage subsets and extracellular vesicles (such as exosomes) in fracture healing have not been fully explored. Additionally, although we established a mouse femoral fracture model to mimic the *in vivo* environment, there are still certain differences between the mouse model and the physiological and pathological processes of human fracture healing. Future clinical studies are needed to validate the findings of this study.

In conclusion, this study has uncovered the crucial mechanism through which migrasomes derived from Tspan4^+^Mpeg1^+^ macrophages facilitate the migration of MSCs via the AMPK pathway within the microenvironment of fracture healing. This research not only provides novel insights into the cell - cell communication mechanisms underlying bone regeneration but also offers potential targets and directions for the research and development of fracture treatment strategies.

## Data Availability

The original contributions presented in the study are included in the article/supplementary material, further inquiries can be directed to the corresponding authors.

## References

[B1] Bayly-Jones C. Pang S. S. Spicer B. A. Whisstock J. C. Dunstone M. A. (2020). Ancient but not forgotten: new insights into MPEG1, a macrophage perforin-like immune effector. Front. Immunol. 11, 581906. 10.3389/fimmu.2020.581906 33178209 PMC7593815

[B2] Chen S. Yu Y. Xie S. Liang D. Shi W. Chen S. (2023). Local H (2) release remodels senescence microenvironment for improved repair of injured bone. Nat. Commun. 14, 7783. 10.1038/s41467-023-43618-z 38012166 PMC10682449

[B3] Jiang F. Zhao H. Zhang P. Bi Y. Zhang H. Sun S. (2024). Challenges in tendon-bone healing: emphasizing inflammatory modulation mechanisms and treatment. Front. Endocrinol. (Lausanne) 15, 1485876. 10.3389/fendo.2024.1485876 39568806 PMC11576169

[B4] Jiao H. Li X. Li Y. Guo Z. Yang Y. Luo Y. (2024). Packaged release and targeted delivery of cytokines by migrasomes in circulation. Cell Discov. 10 (1), 121. 10.1038/s41421-024-00749-x 39648224 PMC11625823

[B5] Justynski O. Bridges K. Krause W. Forni M. F. Phan Q. M. Sandoval-Schaefer T. (2023). Apoptosis recognition receptors regulate skin tissue repair in mice. Elife 12, e86269. 10.7554/eLife.86269 38127424 PMC10735221

[B6] Kim J. M. Lin C. Stavre Z. Greenblatt M. B. Shim J. H. (2020). Osteoblast-osteoclast communication and bone homeostasis. Cells 9 (9), 2073. 10.3390/cells9092073 32927921 PMC7564526

[B7] Xu X. Wu T. Lin R. Zhu S. Ji J. Jin D. (2023). Differences between migrasome, a 'new organelle', and exosome. J. Cell Mol. Med. 27 (23), 3672–3680. 10.1111/jcmm.17942 37665060 PMC10718147

[B8] Yu X. Hu Y. Lim H. Y. Li Z. Jaitin D. A. Yang K. (2025). Septal LYVE1(+) macrophages control adipocyte stem cell adipogenic potential. Science 389, eadg1128. 10.1126/science.adg1128 40875853

[B9] Zhang S. Kutibiding A. Wang M. Zhou X. Tian X. Liu D. (2025). M2 macrophage-derived migrasomes orchestrate dual CXCL12/CXCR4 and neutrophil-MMP-9/MSC-EphB2 signaling to enhance fracture healing. Cell Biomater., 100258. 10.1016/j.celbio.2025.100258

[B10] Zhang X. Yao L. Meng Y. Li B. Yang Y. Gao F. (2023). Migrasome: a new functional extracellular vesicle. Cell Death Discov. 9 (1), 381. 10.1038/s41420-023-01673-x 37852963 PMC10584828

[B11] Zhao X. Lei Y. Zheng J. Peng J. Li Y. Yu L. (2019). Identification of markers for migrasome detection. Cell Discov. 5, 27. 10.1038/s41421-019-0093-y 31123599 PMC6527679

